# Mechanosensitivity during lower extremity neurodynamic testing is diminished in individuals with Type 2 Diabetes Mellitus and peripheral neuropathy: a cross sectional study

**DOI:** 10.1186/1471-2377-10-75

**Published:** 2010-08-28

**Authors:** Benjamin S Boyd, Linda Wanek, Andrew T Gray, Kimberly S Topp

**Affiliations:** 1 Assistant Professor at Samuel Merritt University, Department of Physical Therapy, 450 30th Street, Oakland, CA 94609, USA; 2 Professor and Director of Physical Therapy at San Francisco State University, Department of Physical Therapy, 1600 Holloway, Gym 105, San Francisco, CA 94132, USA; 3 Associate Professor in Residence at the University of California, San Francisco, Department of Anesthesia, Box 1371, 1001 Potrero Ave, San Francisco General Hospital, CA. 94143-1371, USA; 4 Professor and Director of Physical Therapy at the University of California, San Francisco, Graduate Program in Physical Therapy, 1318 7th Avenue, Box 0736, San Francisco, CA 94143-0736, USA; 5 Professor in the Department of Anatomy, Box 0452, San Francisco, CA 94143-0452, USA

## Abstract

**Background:**

Type 2 Diabetes Mellitus (T2DM) and diabetic symmetrical polyneuropathy (DSP) impact multiple modalities of sensation including light touch, temperature, position sense and vibration perception. No study to date has examined the mechanosensitivity of peripheral nerves during limb movement in this population. The objective was to determine the unique effects T2DM and DSP have on nerve mechanosensitivity in the lower extremity.

**Methods:**

This cross-sectional study included 43 people with T2DM. Straight leg raise neurodynamic tests were performed with ankle plantar flexion (PF/SLR) and dorsiflexion (DF/SLR). Hip flexion range of motion (ROM), lower extremity muscle activity and symptom profile, intensity and location were measured at rest, first onset of symptoms (P1) and maximally tolerated symptoms (P2).

**Results:**

The addition of ankle dorsiflexion during SLR testing reduced the hip flexion ROM by 4.3° ± 6.5° at P1 and by 5.4° ± 4.9° at P2. Individuals in the T2DM group with signs of severe DSP (n = 9) had no difference in hip flexion ROM between PF/SLR and DF/SLR at P1 (1.4° ± 4.2°; paired t-test p = 0.34) or P2 (0.9° ± 2.5°; paired t-test p = 0.31). Movement induced muscle activity was absent during SLR with the exception of the tibialis anterior during DF/SLR testing. Increases in symptom intensity during SLR testing were similar for both PF/SLR and DF/SLR. The addition of ankle dorsiflexion induced more frequent posterior leg symptoms when taken to P2.

**Conclusions:**

Consistent with previous recommendations in the literature, P1 is an appropriate test end point for SLR neurodynamic testing in people with T2DM. However, our findings suggest that people with T2DM and severe DSP have limited responses to SLR neurodynamic testing, and thus may be at risk for harm from nerve overstretch and the information gathered will be of limited clinical value.

## Background

Diabetes mellitus (DM) is a group of metabolic disorders that are characterized by hyperglycemia [1]. The Center for Disease Control (CDC) estimated the mean prevalence of DM in the United States (US) in 2005 was 7.0% [2]. Type 2 diabetes mellitus (T2DM) is the most common form of diabetes and is estimated to represent 90-95% of those in the US with diabetes [2]. In 2002, the total estimated direct and indirect costs of DM medical care in the US was $132 billion [3].

Chronic hyperglycemia has adverse metabolic and vascular consequences for the peripheral nervous system [1,4]. Distal symmetrical polyneuropathy (DSP) is the most common neural consequence of hyperglycemia and is present in 30%-60% of people with T2DM depending on the methodology for assessment [1,5,6]. DSP presents as distal, symmetrical sensory alterations that begin in the feet and progress into the legs and hands [1,5-8]. Multiple types of sensation are affected in DSP including vibration sense [9,10], light touch sensation [6,8], position sense [5,8], temperature discrimination [5,7,8], as well as diminished ankle reflexes [5-8]. Pain can also be present [1,5-8]. Motor loss is usually minor or sub-clinical until advanced stages of the disease [1,5-7]. The severity of DSP is related to the duration and severity of hyperglycemia [6,7].

Clinical neurodynamic tests are procedures designed to assess the mechanosensitivity of the nervous system through sequential limb movements [11-14]. Multiple studies have examined the response of neural structures in the lower extremity to a straight leg raise test (SLR) in people with healthy nervous systems [12,13,15-17]. In these studies, ankle dorsiflexion is most commonly used to "sensitize" the SLR test by adding longitudinal loads to the sciatic and tibial nerves. No study to date has examined the mechanosensitivity of peripheral nerves to the elongation and compression associated with limb movement in people with T2DM. In fact, most studies examining neurodynamic testing specifically exclude people with T2DM [12,14,18-20]. Since T2DM and DSP have been shown to affect multi-modal sensory, reflex and motor systems in the distal lower extremities [1,5-8], we expected to find a similar reduction in peripheral nerve mechanosensitivity. The objective of this study was to determine the unique effects of T2DM and DSP severity on peripheral nerve mechanosensitivity in the lower extremity to enhance our understanding of appropriate activity guidelines and physical assessment considerations for people with T2DM. We present the unique impacts of T2DM and DSP on range of motion changes, muscle activity, and symptoms during SLR neurodynamic testing.

## Methods

This cross sectional study included 43 people with T2DM recruited from local medical and academic facilities. Sample size was based on power calculations for a multiple linear regression model with 5 predictor variables, alpha = 0.05, power of 0.8, and an effect size = 0.35. Exclusion criteria included low back or leg pain lasting >3 consecutive days in the past 6 months, complex regional pain syndrome, lumbar spine surgeries, chemical dependence or alcohol abuse, a history of sciatica or trauma to the nerves of the lower extremity, or chemotherapy in the past year. Participants had to meet flexibility requirements of isolated hip flexion ≥90°, full knee extension, ankle dorsiflexion ≥0° and plantar flexion ≥30°. Institutional review boards at UCSF, SFSU and the General Clinical Research Center's Advisory Committee at UCSF approved the study and assured compliance with the ethical treatment of human subjects. Written informed consent was obtained from subjects prior to testing. All subjects attended a single clinical assessment session. One examiner (BB) performed all physical examinations.

### Clinical assessments

#### Questionnaires

Participants completed 1) a medical history questionnaire, 2) the Brief Pain Inventory - Short Form (BPI-SF) [21], 3) the Michigan Neuropathy Screening Instrument questionnaire (MNSIq), and 4) the Modified Baecke Questionnaire (MBQ). The MNSIq has 15 questions addressing symptoms associated with neuropathy [22]. The MBQ is a self-report questionnaire on physical activity during work, recreation/sport, and leisure time [23].

#### Vibration sensory testing

Vibration perception thresholds (VPT) were measured bilaterally on the distal pad of the halluces using a 60-Hz biothesiometer (Bio-Medical Instrument Company, Newbury, OH). The halluces were tested in prone with the leg supported in 90° knee flexion and neutral ankle. The tip of the biothesiometer was balanced on the testing site to ensure consistent contact pressure as described previously [24,25]. The voltage was increased at variable speeds to a maximum of 50 V then reduced to 0 V position to avoid subject anticipation. The subject was instructed to report the first feeling of vibration (VPT). Measurements were repeated twice on each limb and averaged. A bilateral VPT average was calculated (VPT-AVG).

#### Clinical neuropathy examinations

Two scoring instruments of composite physical examinations were used as additional means of quantifying severity of DSP [22]. First, the Michigan Neuropathy Screening Instrument clinical examination (MNSIc) was performed, which included visual inspection for foot deformities or ulcerations, ankle deep tendon reflexes, tuning fork vibration perception (128-Hz) and monofilament sensory testing (10-gram) of the dorsal halluces [22]. Scoring for each examination has been described in detail [22,26,27]. Briefly, ankle reflexes were scored as present, present with reinforcement or absent. Reinforcement consisted of looking away from the testing side and gripping hands and pulling apart. The subject's perception of tuning fork vibration cessation was scored as present (<10-second discrepancy between subject and tester), diminished (≥10-seconds discrepancy) or absent (unable to feel). Monofilament testing was scored as normal (≥8/10 correct responses), decreased (<8/10 correct responses), or absent (unable to feel).

Second, the Michigan Diabetic Neuropathy Score (MDNS) clinical examination was performed, which included Achilles, patellar, biceps brachii and triceps brachii deep tendon reflexes, monofilament, tuning fork vibration and sharp/dull sensation of the dorsal halluces and muscle strength of finger abduction, ankle dorsiflexion and halluces extension. Scoring for each examination has been described in detail [22,26,27]. Reflexes, vibration and monofilament sensation were scored identically to the MNSIc. Reinforcement for the upper extremity reflexes consisted of looking away while pressing the feet together and lightly clenching the jaw. Sharp/dull sensation was scored as present (≥3/5 correct responses) or absent (<3/5 correct responses). Muscle strength was assessed with manual resistance and scored according to the method of Feldman et al. as normal strength, mild to moderate weakness, severe weakness or inability to perform the test (complete loss) [22].

### Straight Leg Raise (SLR) Testing

#### Electromyography

Standard surface electromyography (EMG) electrodes were placed over the soleus, medial gastrocnemius, tibialis anterior, vastus medialis, rectus femoris, semitendinosus, biceps femoris, and gluteus maximus muscles of the right lower extremity [12]. Skin preparation and location of electrode placement was in accordance with the surface electromyography for non-invasive assessment of muscles (SENIAM) guidelines [28]. A single reference electrode was placed over the ipsilateral patella. Each subject performed three repetitions of maximal voluntary isometric muscle contractions (MVC) for 5 seconds each for purposes of EMG signal normalization [12,29,30]. Manual resistance was provided by the tester with the subject in supine and the limb stabilized immediately proximal to the joint being tested. Instructions were given to either push or pull against the examiner's resistance and to not let the examiner move the limb. The calf musculature was tested in a neutral ankle position, the quadriceps and hamstrings were tested with the knee in approximately 30° flexion, and the gluteal musculature was tested in approximately neutral hip position [12,29,30]. EMG signals were acquired with a bandwidth frequency of 50-500 Hz and amplified (2000×) at a sampling rate of 2000 Hz using a TeleMyo 900 System, NorBNC and an A/D USB converter using MRXP Master Package software, Version 1.06.21 (Noraxon USA Inc., Scottsdale, AZ).

#### Goniometry

Twin-axis electrogoniometers (Noraxon USA Inc, Scottsdale, AZ) were used to measure sagittal and coronal plane movement of the right hip and knee [12]. The goniometers were placed laterally over the joint line aligned with the subject's proximal and distal segments for both the hip and knee. Goniometers were held in place with double-sided tape and custom made neoprene straps. Coronal plane motions for the hip and sagittal plane motions for the knee were used to confirm that neutral hip abduction and adduction and full knee extension were maintained during SLR testing. Subjects utilized a custom-built hand held electronic button (trigger) held in their dominant hand to indicate timing of symptoms at start, onset of symptoms (P1) and maximally tolerated symptoms (P2) [12]. Data from these devices were acquired at 2000 Hz and synchronized with the EMG data using the NorBNC and A/D USB converter (Noraxon USA Inc., Scottsdale, AZ).

#### Test procedure

Straight leg raise (SLR) neurodynamic testing methodology has been described [12]. In brief, the subject was positioned in supine with standardized head support. Additional pillows were provided if requested. The subject's right ankle was placed in an APU^® ^PRAFO^® ^ankle brace with outrigger bar and extra straps (Anatomical Concepts, Inc, Poland, Ohio) to maintain a fixed ankle position in either plantar flexion (30°) for the base SLR test (PF/SLR) or in neutral (0°) dorsiflexion for the "sensitized" SLR test (DF/SLR) [12]. One instructional trial was performed on the left lower extremity prior to testing the right lower extremity. Testing on the right extremity included a total of 4 SLR tests, with 2 trials, assigned in a random order, for each ankle position.

The tester placed the subject's knee in full extension (defined as end range resistance) and the subject was instructed to indicate this start position (START) by pressing the hand held trigger. Maintaining this knee position, the subject's hip was moved passively into flexion at a rate of approximately 3°/sec while manually avoiding hip rotation, abduction, or adduction. During this passive hip flexion movement, the subject pressed the hand held trigger to identify the moment they first felt the onset of ANY symptoms (P1) and when their symptoms became too intense to continue and they felt they could not tolerate any further movement (P2). The SLR test was stopped at P2 and this position was held for 5 seconds before the limb was returned to resting on the plinth. Two-minute rests were given between each SLR trial. Subjects were asked to report their symptoms including location, intensity, and quality at each of these time points; START, P1, P2, and after 2 minutes rest.

#### Data processing

Mean voltage for EMG and degrees for hip range of motion were obtained for a 100 msec window centered on each of the following 4 time points; relaxed pre-testing, START, P1, and P2. Raw EMG signals were converted using a root mean squared (RMS) formula with a 50 msec interval and then normalized using the average from the center 3-second window of the three trials of MVC testing (presented as %MVC) [12,30]. A "triggered muscle response" was defined as an increase in EMG activity (expressed as %MVC) of at least a 1.5 fold above resting levels in the "relaxed pre-testing" position taken lying supine prior to establishing the START position [12]. SLR speed was calculated in degrees per second from START position to P2.

### Laboratory Testing

Subjects underwent a blood draw at the General Clinical Research Center at UCSF. Blood samples were sent to Quest Diagnostics, Inc (San Jose, CA) for hemoglobin A1c (HbA1c) and mean plasma glucose (MPG) analysis. These laboratory tests estimate average blood glucose levels over the preceding 2-3 months.

### Statistical Analysis

SPSS software, version 16.0 (SPSS Inc, Chicago, IL) was used to perform all statistical analyses. Descriptive statistics were used to describe the means ± standard deviations (SD) for all variables except frequency descriptive statistics for symptom quality and location, which are reported as percentages. Repeated measures reliability analysis included intraclass correlation coefficients (ICC 3,1) including 95% confidence intervals (lower bound, higher bound). Relationships between demographic, clinical measures and SLR testing variables were assessed using Pearson correlation coefficients. Repeated measures general linear models were used for within test differences between the START, P1 and P2 positions for EMG and ROM data. Paired t-tests were used for between test comparisons (DF/SLR to PF/SLR). One-way ANOVA was used to determine the influence of DSP measures on EMG and ROM data using subgroups defined by severity of neuropathy. Subgroups were based on the bilateral averaged VPT (VPT-AVG): Group 1) no signs of DSP: 0-15 V; Group 2) signs of mild DSP: >15-25 V; Group 3) signs of moderate DSP: >25-50 V; and Group 4) signs of severe DSP: >50 V. The thresholds for Groups 1-3 are based on the relative risk of developing foot ulcerations as outlined in a literature review by Garrow and Boulton [9]. Group 4 is based on the finding that there is a more severe level of DSP unique from the other groups that can be characterized as individuals unable to feel the vibration of the biothesiometer at the 50 V ceiling measurement [25]. Symptom intensity was tested with non-parametric statistics, including Kruskal-Wallis tests for independent comparisons and Friedman's test for related comparisons due to non-normal distributions. Alpha was set at 0.05.

## Results

The sample of people with T2DM (n = 43) consisted of 21 females and 22 males with an average age of 56.3 ± 11.1 (range 21-75) years. Demographic information and clinical examinations of signs of neuropathy included VPT, MNSIc, and MDNS are provided in Table 1. Vibration perception thresholds (VPT) repeated measures reliability (ICC) was 0.97 (95% CI, 0.96, 0.98). Mean MNSIc scores of 3.9 ± 2.4 exceeded the ≥2 cutoff for identifying DSP [31], and mean MDNS of 13.8 ± 8.7 exceeded the >6 cutoff for identifying DSP [22]. Subgroup analysis showed that MDNS scores extended over the range of severity of DSP (p < 0.001). Specifically, those individuals with signs of severe DSP had the highest scores on the MDNS (22.4 ± 8.3) compared to individuals with no signs of DSP (4.6 ± 5.0, p < 0.001), individuals with signs of mild DSP (8.8 ± 4.4, p < 0.001) and individuals with signs of moderate DSP (16.5 ± 5.8, p = 0.023).

**Table 1:** Demographic, clinical measures and correlations

**Demographics**		**Correlations**								
	
		**Subgroup**	**Age**	**BMI**	**HbA1c**	**MDNS**	**MNSIc**	**MNSIq**	**VPT-AVG**	**ModBaecke**
		
Subgroup		-----	0.59*	-0.08	0.08	0.74*	0.69*	0.19	0.92*	-0.18
Age (years)	56.3 ± 11.1	0.59*	-----	0.08	-0.08	0.29	0.43*	-0.06	0.58*	0.01
Height (m)	1.7 ± 0.1									
Weight (kg)	94.0 ± 18.3									
BMI	32.8 ± 6.6	-0.08	0.08	-----	0.43*	0.04	0.35*	0.36*	-0.06	-0.28
Duration of T2DM (years)	7.0 ± 7.7									
Gender	49% female/51% male									
HbA1c	7.4 ± 1.8	0.08	-0.08	0.43*	-----	0.24	0.35	0.28	0.12	-0.57*
										
**Vibration perception threshold (VPT)**										
Right halluces (volts)	30.1 ± 14.8									
Left halluces (volts)	29.9 ± 15.6									
VPT-AVG for bilateral halluces (volts)	30.0 ± 14.9	0.92*	0.58*	-0.06	0.12	0.75*	0.71*	0.27	-----	-0.20
										
MNSIq (0-13)	3.8 ± 2.4	0.19	-0.06	0.36*	0.28	0.39*	0.57*	-----	0.27	-0.34*
MNSIc (0-10)	3.9 ± 2.4	0.69*	0.43*	0.35*	0.35	0.80*	-----	0.57*	0.71*	-0.28
MDNS (0-46)	13.8 ± 8.7	0.74*	0.29	0.04	0.24	-----	0.80*	0.39*	0.75*	-0.19
										
**ModBaecke Questionnaire**										
Work subscale	2.5 ± 0.5									
Sports subscale	2.7 ± 1.1									
Leisure subscale	2.7 ± 0.5									
Total score	7.9 ± 1.7	-0.18	0.01	-0.28	-0.57*	-0.19	-0.28	-0.34*	-0.20	-----

Abbreviations: Body mass index (BMI), hemoglobin A1c (HbA1c), Michigan diabetic neuropathy score (MDNS), Michigan neuropathy screening instrument - clinical portion (MNSIc), Michigan neuropathy screening instrument - questionnaire portion (MNSIq), Vibration perception threshold averaged for right and left halluces (VPT-AVG), and the Modified Baecke questionnaire total score (ModBaecke).

Pearson product correlations are presented with significance set at p < 0.05 (*)

Correlations between assessment tools and demographic data are presented in Table 1. The three clinical measurement tools used to identify signs of DSP (VPT, MDNS, MNSIc) were highly correlated (>0.70 correlation coefficient). The strongest such correlations were between MDNS and MNSIc (0.82, p < 0.0005) and between MDNS and VPT-AVG (0.76, p < 0.0005). Subgroups correlated with age (0.59, p < 0.001), MDNS score (0.74, p < 0.001), MNSIc score (0.69, p < 0.001). Age had a high correlation with VPT-AVG (0.57, p < 0.0005) but did not correlate with maximal hip flexion ROM at P2 for PF/SLR (-0.14, p = 0.370).

The clinical questionnaires for symptoms related to neuropathy (MNSIq) and activity level (Modified Baecke questionnaire) are presented in Table 1. Further analysis revealed that there were no differences between MNSIq scores amongst subgroups (p = 0.480). The Modified Baecke questionnaire work, sports and leisure subscales and the total score did not vary amongst subgroups (p = 0.659, p = 0.347, p = 0.516, p = 0.324, respectively). From the BPI-SF, the reported average pain rating (Q5) was 3.0 ± 2.6 on a 0-10 point scale. The reported pain "right now" (Q6) was 1.9 ± 2.2 on a 0-10 point scale. Subgroup analysis revealed that there was no difference in Q5 ratings (p = 0.894) or Q6 ratings (p = 0.762) between subgroups.

### Straight leg raise neurodynamic testing

The average speed of hip flexion during PF/SLR was 2.3 ± 1.2°/second and DF/SLR was 2.1 ± 1.2°/second (p = 0.002). Amongst the participants, 23.3% did not request any extra head support, 65.1% requested 1 pillow, 9.3% requested 2 pillows and 2.3% requested 3 pillows during the SLR testing. The number of pillows did not affect the hip range of motion for either DF/SLR or PF/SLR at either P1 or P2 (p = 0.55-0.94).

#### Range of motion

**Figure 1:**
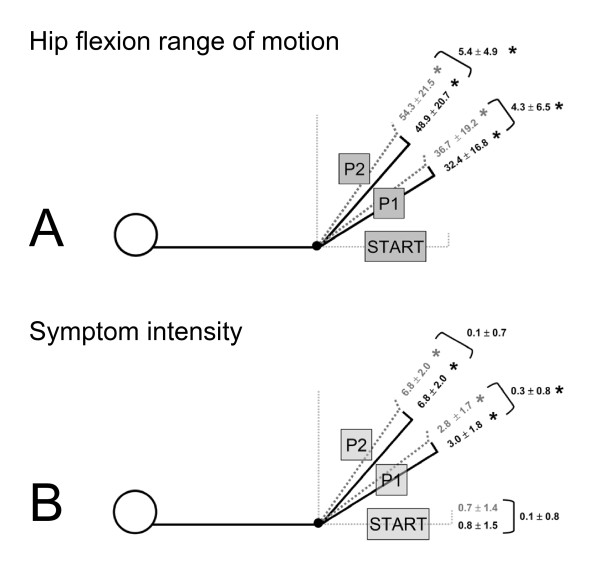
**Range of motion and symptom intensity during SLR neurodynamic testing**. Straight leg raise neurodynamic test results are presented for hip flexion range of motion in degrees (**A**) and symptom intensity (0-10 scale) (**B**). Gray dotted lined body diagrams represents the PF/SLR test and black lined body diagrams represent the DF/SLR test. Significance is indicated by an * and was set at p < 0.05. **A) **Hip flexion to the onset of symptoms (**P1**) and to the maximally tolerated position (**P2**) is significantly greater than the zeroed **START **position (p < 0.05). The differences between the PF/SLR and the DF/SLR are 4.3° at P1 and 5.4° at P2 (p < 0.05). **B) **Symptom intensity on a 0-10 point scale is presented for each SLR. The gray dotted line represents the PF/SLR test and the black line represents the DF/SLR test. **START **position is full manual knee extension prior to hip flexion while supine lying. **P1 **represents the moment of first onset of symptoms or the first increase above resting symptoms and **P2 **represents the maximally tolerated symptom position. Symptom intensity is significantly increased at both P1 and P2 over START position values (p < 0.05). There was 0.3-point significantly greater symptom intensity at P1 when in the DF/SLR test compared to the PF/SLR test.

ICCs for hip flexion range of motion (ROM) between trials were 0.90 (95% CI, 0.82, 0.94) for PF/SLR at P1, 0.94 (95% CI, 0.90, 0.97) for PF/SLR at P2, 0.89 (95% CI, 0.80, 0.94) for DF/SLR at P1 and 0.96 (95% CI, 0.92, 0.98) for DF/SLR at P2. The hip range of motion to P1 and to P2 during SLR was greater than the START position for both DF/SLR and PF/SLR (p < 0.0005) (Figure 1A). There was 4.3° ± 6.5° less hip flexion ROM at P1 during DF/SLR compared to PF/SLR (P1diff) (p < 0.001) (Figure 1A). At P2 there was 5.4 ± 4.9° less hip flexion ROM during DF/SLR compared to PF/SLR (P2diff) (p < 0.0005) (Figure 1A).

**Figure 2:**
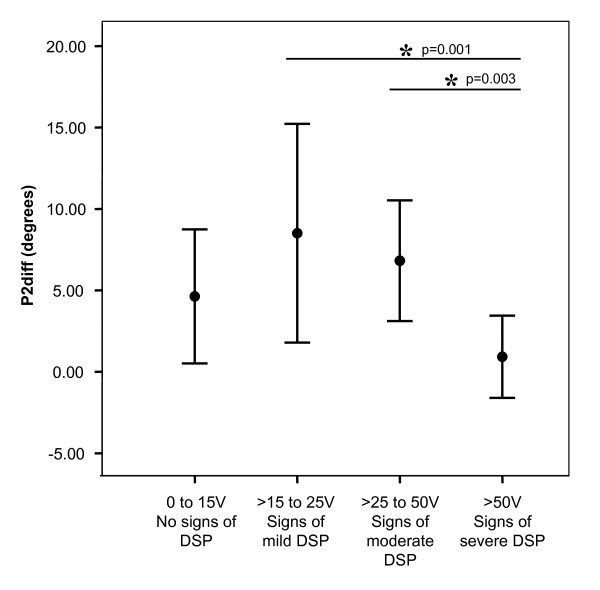
**Impact of DSP severity subgroups on difference in range of motion between SLR tests**. The difference in hip flexion range of motion between PF/SLR and DF/SLR at the maximally tolerated symptom position (P2) is presented on the Y-axis (P2diff) in degrees. Subgroups are presented on the X-axis and include the following subgroups based on bilateral averaged VPT (VPT-AVG); 1) no signs of DSP: 0-15 V, 2) signs of mild DSP: >15-25 V, 3) signs of moderate DSP: >25-50 V, and 4) signs of severe DSP: >50 V. Individuals with signs of severe DSP had a significantly lower P2diff compared to two of the other subgroups. Significance is indicated by an * and was set at p ≤ 0.05.

Further analysis revealed no effect of subgroup on hip flexion range of motion for the P1diff measure. However, those individuals with signs of severe DSP had no statistical difference in hip flexion ROM between PF/SLR and DF/SLR tests (1.4° ± 4.2°; paired t-test p = 0.34). There was a significant effect of subgroup on the difference in hip flexion ROM between PF/SLR and DF/SLR at P2 (P2diff, p = 0.005) (Figure 2). The P2diff measure was 0.9° ± 2.5° in individuals with signs of severe DSP compared to 6.6° ± 4.7° in the remainder of the participants (all other subgroups combined). The eta value for VPT-AVG subgroups was 0.53 with an eta^2 ^of 0.28, which indicates that subgroup explains 28% of the variability in the P2diff outcome measure. There was 1.9° ± 4.5° more hip abduction at P2 during PF/SLR compared to DF/SLR (p = 0.008). There were no differences in hip abduction at P1 between PF/SLR and DF/SLR, nor in knee extension or valgus at either P1 or P2 (p = 0.13-0.96, data not shown).

#### Muscle activation

Muscle activity during SLR testing is presented in Table 2. Using the conservative threshold of 1.5-fold increase over resting levels, no muscle activity was triggered at the START position, P1 or P2 during PF/SLR. The addition of ankle dorsiflexion created a small change in the pattern of muscle activation. During the DF/SLR, activity in the tibialis anterior was triggered when the limb was placed in the START position when the ankle was held in 0° dorsiflexion and the knee was held in full extension. The mean increase in activity was almost a 2-fold increase over resting levels from 5.3 ± 4.1%MVC to 9.7 ± 8.9%MVC. This increased activity was then stable through the remainder of the DF/SLR testing and did not change further when the limb was moved to P1 or to P2.

**Table 2:** Muscle activation pattern (%MVC) during SLR neurodynamic testing

		**PF/SLR**			**DF/SLR**		
		
**Muscle**	**Resting**	**Start**	**P1**	**P2**	**Start**	**P1**	**P2**
						
Soleus	20.2 ± 16.1	19.9 ± 15.7	21.3 ± 16.6†	21.7 ± 16.3	20.7 ± 17.4	23.0 ± 19.5†	22.6 ± 16.7
Medial gastrocnemius	14.6 ± 13.6	16.0 ± 15.2	18.9 ± 19.3	20.2 ± 21.5	16.8 ± 16.2	19.4 ± 20.77	19.9 ± 18.0
Tibialis anterior	5.3 ± 4.1	5.2 ± 4.3†	5.7 ± 5.5†	6.3 ± 6.9†	9.7 ± 8.9 *†	9.0 ± 8.0 *†	9.4 ± 10.1 *†
Vastus medialis	13.8 ± 12.2	13.9 ± 12.9†	16.3 ± 14.9	16.5 ± 14.0	9.9 ± 7.6†	15.7 ± 14.1	15.6 ± 12.9
Rectus femoris	11.8 ± 7.8	12.4 ± 10.1	14.8 ± 14.7	15.9 ± 13.1†	12.6 ± 9.7	13.4 ± 9.9	14.2 ± 9.6†
Semitendinosus	9.3 ± 8.3	9.7 ± 9.0	10.6 ± 9.0	12.4 ± 9.1	9.6 ± 9.5	11.3 ± 9.5	12.7 ± 9.9
Biceps femoris	9.3 ± 6.1	10.4 ± 7.1†	11.2 ± 7.9	13.5 ± 8.8	9.1 ± 6.3†	13.3 ± 17.4	13.4 ± 9.4
Gluteus maximus	28.4 ± 21.0	28.2 ± 23.3	31.6 ± 26.9	36.0 ± 29.2	28.8 ± 22.6	34.8 ± 43.8	38.9 ± 36.1

* denotes statistically significant increase of greater than 1.5x "Resting" levels for general linear model of repeated measures for within test differences.

† denotes statistically significant difference between PF/SLR and DF/SLR tests using paired t-test comparison for "Start" and "P1" and "P2."

Between tests comparisons identified a significantly higher tibialis anterior muscle activation at the START position during DF/SLR compared to PF/SLR (p < 0.001) (Table 2). Interestingly, there was a simultaneous decrease in muscle activation in the vastus medialis and biceps femoris (p < 0.001 and p = 0.032). At P1, there was a significantly higher activation in the tibialis anterior and soleus during DF/SLR compared to PF/SLR (p < 0.001 and p = 0.033). A similar pattern was also present at P2, where there was a significantly higher activation of tibialis anterior during DF/SLR compared to PF/SLR (p = 0.001). Further analyses of muscle activity showed that there was no subgroup effect on muscle activity at any time point; START, P1, or P2.

#### Symptom intensity

The mean symptom intensity at the START position was 0.7 ± 1.4 during PF/SLR (Figure 1B). The symptom intensity increased to 2.8 ± 1.7 at P1 and to 6.8 ± 2.0 at P2 during the PF/SLR (p < 0.001 both). During DF/SLR the mean intensity went from 0.8 ± 1.5 at the START position to 3.0 ± 1.8 at P1 and to 6.8 ± 2.0 at P2 (p < 0.001 both). The mean intensity at P1 was significantly higher by 0.3 ± 0.8 points during DF/SLR compared to PF/SLR (p = 0.043). There was no difference in mean intensity between PF/SLR and DF/SLR at the START position, P2 or rest after SLR. Further analyses revealed no subgroup effect on symptom intensity during PF/SLR or DF/SLR at either P1 or P2.

#### Symptom location

**Figure 3:**
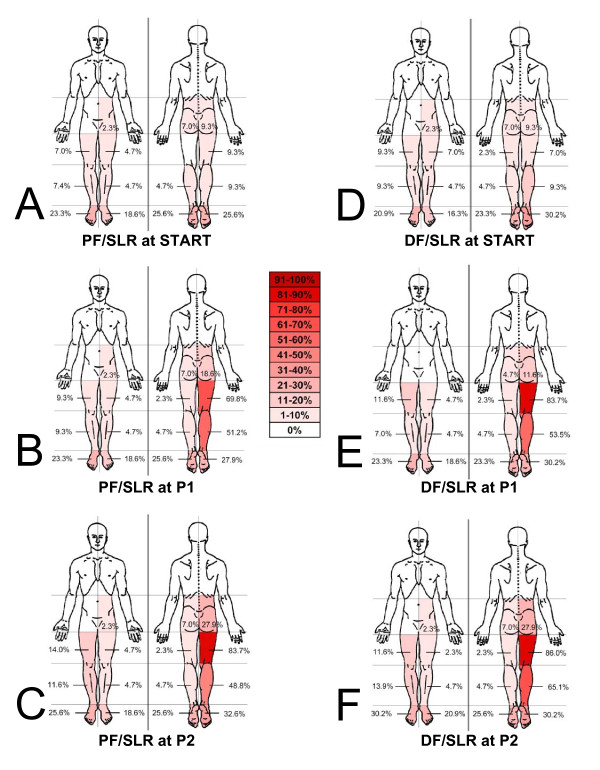
**Reported symptom locations during SLR neurodynamic testing**. Body chart representations are presented for frequencies of symptom location reported during SLR testing. **A, B, C **represents PF/SLR at the START, onset of symptoms (P1) and the maximally tolerated position (P2), respectively. **D, E, F **represents DF/SLR at the START, onset of symptoms (P1) and the maximally tolerated position (P2), respectively. Frequencies are reported in 10% intervals from a white color of 0% frequency to 90-100% as dark red (see key in center of figure). There were >15% bilateral symptoms in the feet at the START position for both PF/SLR and DF/SLR. There were more frequent right posterior leg symptoms in the DF/SLR test when compared to the PF/SLR at P2.

The frequencies of symptom locations reported at the START, P1 and P2 during SLR are presented in Figure 3. More than 15% of subjects reported symptoms in both the dorsal and plantar surfaces of bilateral feet at the START position for both PF/SLR and DF/SLR (Figure 3A &3D). During PF/SLR at P1 and P2, the most frequent symptom location was reported in the right posterior thigh followed by the right posterior leg (Figure 3B &3C). A similar pattern was seen during DF/SLR, including the right posterior thigh being the most frequent symptom location at P1 and P2, followed by the right posterior leg (Figure 3E &3F). During PF/SLR, there was a 7.0% increase in the frequency of reported symptoms in the right plantar foot while there was an 18.6% increase frequency of symptoms reported in the right posterior hip at P2 compared to the START position. During DF/SLR, there was no change in the frequency of right plantar foot symptoms while there was an 18.6% increase frequency of symptoms reported in the right posterior hip at P2 compared to the START position.

With the addition of ankle dorsiflexion (DF/SLR) there was an increase in the frequency of reported movement induced symptoms (not present at START position) at P1 by 16.2% in the posterior thigh compared to PF/SLR. However, the addition of ankle dorsiflexion only increased the frequency of movement induced symptoms by 2.3% in the posterior leg and reduced the frequency of movement induced symptoms by 2.3% in plantar surface of the foot. When taken to P2, the addition of ankle dorsiflexion increased the frequency of movement induced symptoms in the posterior thigh by 4.6% and the posterior leg by 16.3%, while there was a reduction in movement induced symptoms in the plantar surface of the foot by 7.0%

#### Symptom quality

**Figure 4:**
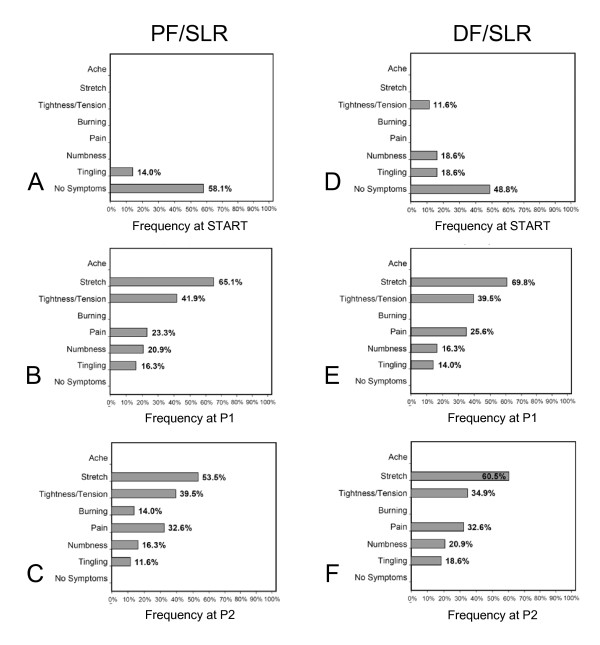
**Reported symptom quality descriptors during SLR neurodynamic testing**. Histograms are presented for frequencies of symptom quality reported during SLR testing. **A, B, C **represents PF/SLR at the START, onset of symptoms (P1), and maximally tolerated position (P2), respectively. **D, E, F **represents DF/SLR at the START, onset of symptoms (P1), and maximally tolerated position (P2), respectively. Symptoms reported in <10% of participants are not presented. The frequency of no symptoms at the START position was greater in the PF/SLR compared to the DF/SLR. The most common symptoms at the START position were numbness and tingling. Stretch and tightness/tension were the two most frequent reported symptoms at P1 and P2 during both SLR tests. Pain was also induced in >20% of the subjects at P1 and >30% of the subjects at P2 during both SLR tests.

Frequencies of symptom descriptions are presented in Figure 4. Symptoms reported in <10% of participants are not presented. At the START position 58.1% of the subjects reported no symptoms during PF/SLR (Figure 4A). The main symptom reported in the START position during PF/SLR was tingling (14.0%). When taken to P1, the additional symptom of stretch was most commonly reported, followed by tightness/tension, then pain, and finally numbness (Figure 4B). When taken to P2, pain frequency increased by 9.3% and burning was reported in 14.0% of the subjects (Figure 4C). In contrast, 48.8% of subjects reported no symptoms at the START position during DF/SLR (Figure 4D). Numbness and tingling were present in 18.6% at the START position during DF/SLR with an additional 11.6% reporting tension/tightness (Figure 4D). When DF/SLR was taken to P1 there was a similar response to PF/SLR where stretch was the most common symptom reported, followed by tightness/tension, and then pain (Figure 4E). When taken to P2 during DF/SLR, the frequency of pain and numbness increased by 7% and 4.6% (Figure 4F).

Between test comparisons revealed that there was only a 2.3% increase in reported pain at P1 during the DF/SLR compared to the PF/SLR and no difference (0%) at P2. There were no differences greater than 5% in frequency of reported stretch, tightness/tension, numbness, tingling between DF/SLR and PF/SLR tests at either P1 or P2 with the one exception being a 7.0% increase in the frequency of reported stretch at P2 during DF/SLR compared to PF/SLR. Further analysis revealed that in individuals with signs of severe DSP, 44.4% (4/9) reported parasthesias (numbness, tingling, pins/needles) at rest prior to beginning testing and 55.6% (5/9) reported them during SLR testing. Of the four subjects that reported paraesthsias at rest, all reported them in bilateral dorsal and plantar surfaces of their feet and these symptoms were unchanged during testing.

## Discussion

We found that mechanosensitivity in the lower extremity is present in people with T2DM when evaluated through clinical neurodynamic testing. However, many of the outcome measures utilized in neurodynamic testing, including changes in hip flexion range of motion with addition of ankle dorsiflexion, symptom location and muscle activity, differed in people with T2DM when compared with previous findings in people without T2DM. Importantly, some of the signs and symptoms that present with the sensitizing maneuver of ankle dorsiflexion were not present in individuals with T2DM with signs of severe neuropathy.

The magnitude of impact that the addition of ankle dorsiflexion has on hip flexion range of motion during SLR testing may reflect the state of mechanosensitivity of the neural structures being tested. Mechanosensitivity is a normal protective response to the stresses applied to nerves during limb movement. Thus, it is reasonable to expect hip flexion range of motion to be reduced when a SLR is performed with the ankle in a position of dorsiflexion. The addition of dorsiflexion has been shown to induce longitudinal gliding and increased strain in the lower extremity posterior neural structures, providing a "sensitized" version of the SLR [32-34]. In previous studies of healthy individuals, the addition of dorsiflexion to the SLR resulted in between 5.5° and 10.1° reduction in SLR angle depending on the test endpoint [12,16]. In people with T2DM we found that the addition of ankle dorsiflexion caused a normal 4° reduction in hip flexion range of motion when tested to P1, but only a 5° reduction when tested to P2. This represents a statistically significant 50% reduction in the effect of dorsiflexion on hip flexion range of motion in people with T2DM compared to people without T2DM when tested to P2 (p = 0.039) [12]. Moreover, in individuals with T2DM and severe DSP the addition of dorsiflexion did not alter hip flexion range of motion at P1 or P2. This represents statistically significant 90% reductions in the effect of dorsiflexion on hip flexion in people with T2DM with severe DSP when tested to P2 compared to people without T2DM (p = 0.001) [12]. The diminished response to the "sensitized" SLR in people with T2DM and severe DSP may reflect a reduced protective response to neural loading during limb movements due to a diminished mechanosensitivity of the lower extremity nervous system.

A comparison to findings in healthy controls in a previous study demonstrates that our sample of people with T2DM had reduced general flexibility during SLR [12]. Specifically, during the SLR with the least load on the nervous system (PF/SLR), people with T2DM had 54.3° ± 21.5° of hip flexion ROM, significantly less than the 67.6° ± 22.1° documented in the same test in healthy individuals without T2DM (p = 0.027) [12]. These findings are in agreement with studies that have documented reduced range of motion in both ankle and hip joints in people with T2DM compared to healthy individuals without DM [35-37]. To our knowledge, our study is the first to document a reduction in available hip range of motion during SLR testing in people with T2DM. Further studies are warranted to examine the mechanisms behind proximal mobility changes in people with T2DM.

The absence of triggered muscle activation found in the present study in people with T2DM is drastically different from that found previously in healthy individuals without T2DM. In the present study we found only the tibialis anterior became activated during the DF/SLR as defined by a 1.5× increase over resting EMG activity. In comparison, a previous study of healthy individuals without T2DM found muscle activation using this same threshold criteria during the PF/SLR in the rectus femoris at P1 and in the soleus, medial gastrocnemius, tibialis anterior, vastus medialis, rectus femoris, biceps femoris, and gluteus maximus at P2 [12]. This study also found activation of the soleus, tibialis anterior, vastus medialis, and semitendinosis muscles during DF/SLR at P1 and the soleus, medial gastrocnemius, tibialis anterior, vastus medialis, rectus femoris, and semitendinosis at P2 [12]. People with T2DM did not exhibit the co-contraction of anterior and posterior limb muscles that was evident in healthy controls at P1 in the sensitized DF/SLR.

Our sample of individuals with T2DM had much higher resting muscle activity in comparison to previously reported healthy individuals without T2DM [12]. It is possible that this apparent higher resting muscle activity is a MVC calculation artifact due to a reduction in contraction effort during MVC procedures either due to true strength deficits or to the presence of resting symptoms. Since resting activity is converted into a %MVC, the values of muscle activity at rest would be higher if the effort during MVC testing was reduced because of the presence of pain. The authors speculate that reduced muscle output during MVC testing due to resting symptoms, contributed to the findings of higher resting activity in our study when expressed as %MVC. One possible explanation for the activation of the tibialis anterior is related to the START position for this DF/SLR test which included fixation in a customized brace. Since the increase in tibialis anterior activity was triggered during dorsiflexion positioning and then remained stable during SLR to P1 or P2, we hypothesize that the brace itself provided pressure or proprioceptive feedback to the participant that created a responsive increase in muscle tone in the tibialis anterior. Further investigation is necessary to elucidate if this finding is clinically important and to understand why this was not observed in the posterior leg musculature.

Reliance solely on muscle activity measures for decisive conclusions for mechanosensitivity is not reasonable due to the high variability of this type of measurement. Some individuals had no muscle activation during SLR testing at P1 and P2, which is consistent with previous study findings [38,39]. In our present study, 12.2% of the people with T2DM had no muscle activity at P2 during PF/SLR compared to 10.0% of healthy individuals without T2DM in our previous study [12]. Comparisons during DF/SLR showed that 14.6% of the people with T2DM had no muscle activity at P2 compared to 5.0% of healthy individuals without T2DM in our previous study [12].

Symptom quality, intensity and distribution differed in people with T2DM when compared to healthy individuals without T2DM [12]. At the START position, people with T2DM frequently reported symptoms associated with neurogenic sources, such as numbness and tingling, which were not present in healthy individuals. During limb movement, people with T2DM reported pain (>20% at P1 and >30% at P2) more frequently than healthy individuals (≤10%). Furthermore, the subgroup of people with signs of severe DSP reported symptoms associated with neurogenic sources, such as numbness and tingling 55.6% of the time and pain 33.3% of the time during SLR testing. The intensity of symptoms reported in people with T2DM during DF/SLR and PF/SLR tests was 0.3 points higher on a 0-10 point numeric pain rating scale at P1. Although this is statistically significant it does not represent a clinically meaningful difference between tests [40]. People with T2DM reported more symptoms at the START position than those without T2DM. The percentage of people with T2DM with symptoms in the START position during PF/SLR testing was 41.9% and during DF/SLR was 51.2%. In comparison, the percentage of healthy individuals without T2DM with symptoms in the START position was 15.0% during PF/SLR and 25.0% during DF/SLR. Lastly, in contrast to healthy controls, people with T2DM reported symptoms on the dorsal and plantar surfaces of bilateral feet in the START position (16-30%) [12]. In healthy individuals without T2DM, frequency of symptoms in the posterior hip was at most 10% compared to 27.9% in people with T2DM, with the most dramatic difference occurring at P2.

In the subgroup of individuals with T2DM and severe DSP we found symptoms and clinical examination signs that were consistent with recent findings examining pain presentation phenotypes in neuropathic pain conditions [41]. Scholz et al. determined eight specific patterns of signs and symptoms of which they found people with DSP presented with three of these patterns [41]. These three patterns had multiple characteristics in common that relate to our findings, including ongoing pain, dysesthesia (primarily tingling and numbness), reduced response to monofilament, pinprick and vibration testing and the absence of a response to passive movement evoked pain and an absent response to straight leg raise testing [41]. These characteristics match the cluster of symptoms and physical signs identified in our study sample, particularly those with signs of severe DSP.

A mechanism that would explain the reduction of mechanosensitivity seen in people with T2DM and DSP has yet to be established. A recent study documented an altered ratio of mechanoresponsive to mechanoinsensitive C-fibers in people with diabetes and mild DSP in the common fibular nerve at the knee level [42]. It was found that the ratio of 2:1 mechanoresponsive:mechanoinsensitive C-fibers in healthy controls without DM or neuropathy was reversed to 1:2 in subjects with DM and mild DSP. The shift appeared to occur simultaneously with a general reduction of C-fibers. The authors concluded that changes in small fiber health associated with DSP leads to an impairment of mechanoresponsive nocioceptors. Although the ratio of mechanoresponsive to mechanoinsensitive C-fibers is unknown in our population of individuals with T2DM and DSP, altered microneurographic findings would be consistent with the decrease in mechanosensitivity during the SLR tests that we found in this population.

One major limitation of our study is the small sample size reducing the power to detect differences between variables with smaller effect sizes and diminishing the ability to extrapolate findings to larger populations. A potential covariate in our study is the impact of age on peripheral nerve health. We attempted to match our sample mean age to previous studies of peripheral nerve health in people with T2DM and studies of SLR neurodynamic testing to allow for valid comparisons. In our study we found no correlation between age and any hip flexion range of motion measure during SLR testing, in agreement with a previous study of age and hamstring length [43]. The average age of our study participants closely resembles larger studies including studies by Fedele et al. examining prevalence in 8757 people in Italy with T2DM (age = 55.8 ± 10.4) and by Rahman et al. examining multiple clinical measures of neuropathy (age = 63.0 ± 7.8) [10,26]. In our study sex was not statistically associated with any hip flexion range of motion outcome measure, although a larger investigation with differing methodology found that women have greater hip range of motion during SLR testing [43].

An additional limitation is the difference of hip abduction range of motion at P2 during PF/SLR compared to DF/SLR which could have influenced the SLR outcome measures. The mean difference was less than 2° between the PF/SLR and DF/SLR and is not likely clinically significant but still represents a potential confounding variable that should be acknowledged in the interpretation of our study findings. Precisely controlled standardized procedures for clinical neurodynamic testing, as were utilized in this study, are warranted to minimize tester induced variability.

One characteristic difference between our present findings and that of SLR neurodynamic test findings in healthy individuals without T2DM is body mass index (BMI). The body mass index was larger in our sample of people with T2DM (32.8 ± 6.6 kg/m^2^) compared to healthy individuals without T2DM (25.9 ± 8.8 kg/m^2^, p = 0.001) [12]. Mean weight in the people with T2DM was also greater in the present study (94.0 ± 18.3 kg) compared to the healthy individuals without T2DM (71.2 ± 24.8 kg, p < 0.001) [12]. Height was not different between these two study samples (p = 0.127) [12]. We did not find a correlation between BMI in people with T2DM and hip flexion range of motion during SLR. We did not measure nutritional deficits in our study such as vitamin B12 deficiencies which may be a potential confounding variable. Future studies should investigate neurodynamic testing in stratified BMI groups, BMI matched groups and vitamin B12 deficiencies to further explore the effects of body mass and nutritional status on mechanosensitivity of peripheral nerves.

Our study supports specific considerations for patient education and therapeutic exercise instructions. Alterations or avoidance of activities that involve cumulative loading of the nervous system via multiple joints should be considered in people with T2DM and signs of DSP. This could include avoiding movements into slumped postures with the feet elevated, altering specific activities of daily living such as forward bending to tie one's shoes, or avoiding specific movements or postures assumed during recreational activities such as yoga or Pilates. An understanding of the health of the nervous system including the ability to respond to and protect against over stretch should be incorporated into clinical decision making for physical examination, exercise prescription and patient education in people with T2DM. Although this study only examined passive limb positioning for short duration, it provides evidence to help support our clinical decisions related to protection of the peripheral nervous system in people with T2DM and DSP.

## Conclusions

We have provided evidence that the normal protective responses to neural loading during neurodynamic testing may be diminished in people with T2DM particularly those with signs of severe DSP. Additionally, we found increased frequency of resting symptoms in people with T2DM and increased frequency of reported neurogenic related symptom qualities during SLR testing. We found the addition of ankle dorsiflexion does not induce the same degree of reduction of hip flexion range of motion and the same pattern of muscle protective guarding during SLR testing in people with T2DM. The findings of our study call into question the clinical decision of performing neurodynamic testing on people with signs of severe DSP. Without the ability to respond to the increases in neural loading associated with neurodynamic testing and sensitizing maneuvers, this population is at risk for injury from testing and the information gathered will be of limited use to the clinician. It is recommended that clinicians perform a simple screen of sensation such as vibration perception testing or the MDNS prior to considering the appropriateness of neurodynamic assessments.

When considering testing in people with T2DM, it is important to understand the global impact on mechanosensitivity due to the common distal symmetrical polyneuropathy associated with T2DM. When neurodynamic testing is deemed appropriate in this population, additional considerations are necessary for test interpretation in people with T2DM. It is paramount to clearly establish the person's resting symptoms prior to performing SLR testing. Symptoms that are normally associated with neurogenic sources may be present bilaterally at rest confounding SLR test interpretation in people with T2DM. Consideration of specific movement induced symptoms in addition to symptoms present at rest should assist the clinician interpret neurodynamic testing appropriately. Previous recommendations have included utilizing the onset of symptoms (P1) or the increase above resting symptoms as the end point of the SLR neurodynamic test [12,44]. The findings of our study support using this same end point for SLR testing in people with T2DM to avoid potential harm of over stressing the nervous system [12].

## Competing interests

The authors declare that they have no competing interests.

## Authors' contributions

BSB provided input into study design, performed all clinical testing, performed the statistical analysis and helped to draft the manuscript. LW provided input into study design and helped to draft the manuscript. ATG provided input into study design and helped to draft the manuscript. KST provided input into study design and helped to draft the manuscript. All authors read and approved the final manuscript.

## Pre-publication history

The pre-publication history for this paper can be accessed here:

http://www.biomedcentral.com/1471-2377/10/75/prepub
